# Automatic segmentation of nasopharyngeal carcinoma on CT images using efficient UNet‐2.5D ensemble with semi‐supervised pretext task pretraining

**DOI:** 10.3389/fonc.2022.980312

**Published:** 2022-11-11

**Authors:** Jansen Keith L. Domoguen, Jen-Jen A. Manuel, Johanna Patricia A. Cañal, Prospero C. Naval

**Affiliations:** ^1^ Computer Vision and Machine Intelligence Group, Department of Computer Science, University of the Philippines-Diliman, Quezon City, Philippines; ^2^ Division of Radiation Oncology, Department of Radiology, University of the Philippines-Philippine General Hospital, Manila, Philippines

**Keywords:** nasopharyngeal carcinoma, automatic volume segmentation, deep learning, radiotherapy, semi-supervised learning, pretext tasks

## Abstract

Nasopharyngeal carcinoma (NPC) is primarily treated with radiation therapy. Accurate delineation of target volumes and organs at risk is important. However, manual delineation is time-consuming, variable, and subjective depending on the experience of the radiation oncologist. This work explores the use of deep learning methods to automate the segmentation of NPC primary gross tumor volume (GTVp) in planning computer tomography (CT) images. A total of sixty-three (63) patients diagnosed with NPC were included in this study. Although a number of studies applied have shown the effectiveness of deep learning methods in medical imaging, their high performance has mainly been due to the wide availability of data. In contrast, the data for NPC is scarce and inaccessible. To tackle this problem, we propose two sequential approaches. First we propose a much simpler architecture which follows the UNet design but using 2D convolutional network for 3D segmentation. We find that this specific architecture is much more effective in the segmentation of GTV in NPC. We highlight its efficacy over other more popular and modern architecture by achieving significantly higher performance. Moreover to further improve performance, we trained the model using multi-scale dataset to create an ensemble of models. However, the performance of the model is ultimately dependent on the availability of labelled data. Hence building on top of this proposed architecture, we employ the use of semi-supervised learning by proposing the use of a combined pre-text tasks. Specifically we use the combination of 3D rotation and 3D relative-patch location pre-texts tasks to pretrain the feature extractor. We use an additional 50 CT images of healthy patients which have no annotation or labels. By semi-supervised pretraining the feature extractor can be frozen after pretraining which essentially makes it much more efficient in terms of the number of parameters since only the decoder is trained. Finally it is not only efficient in terms of parameters but also data, which is shown when the pretrained model with only portion of the labelled training data was able to achieve very close performance to the model trained with the full labelled data.

## 1 Introduction

Nasopharyngeal carcinoma is rare among Caucasians but one of the more common head and neck cancers found among Asians and North Africans (1). Standard treatment involves combination chemotherapy and radiotherapy. Surgery is generally done as salvage after treatment inadequacies or failures. Over the past few decades and with improved digitalization, radiation therapy has become more and more precise. This came about because of precision in both cross-sectional diagnostic imaging (CT and MRI) and radiation delivery. Precision is the key. In the process of radiotherapy, one of the most critical steps is contouring of the tumor. After all, if the target is incorrect or imprecise in any way, the subsequent treatment planning and treatment delivery will be incorrect and imprecise too.

With the advent of artificial intelligence, there is now software available for auto-contouring. All commercially available treatment planning systems contain software that can auto-contour normal structures or organs. At the present, much research is being done into auto-contouring the gross tumor volume (GTV), many of them coming out of China. Since nasopharyngeal carcinoma is considered endemic in China, it is logical that resources are being poured into creating artificial intelligence that can map nasopharyngeal tumors on CT scans and MRIs.

There are at least 6 studies that have dealt with auto-contouring of nasopharyngeal tumors using cross-sectional imaging, both CT scan and MRI (2–6). Work by (2) was one of the earliest works who applied deep learning methods on the segmentation of NPC. They proposed a modified UNet architecture where the downsampling and upsampling layers have similar number of parameters to ensure that the output resolution is exactly the same as the input. Moreover, their work also analyzed the performance of deep neural networks across different tumors stages as well as predicting gross nodal volumes. They observed significant performance degradation as the tumor stage increases and a much lower performance for gross nodal volumes. In contrast to our work, we don’t distinguish tumor stage for our performance analysis. Work by (3) proposed a novel 3D convolutional network which uses cascaded multi-scale local enhancement for convolutional networks. Specifically they adopted the 3D Res-UNet as their backbone network and employed a multi-scale dilated convolutional block to enhance extracted receptive field and improve focus on the target tumor especially its boundary. This is then integrated to a central localization cascade model to concentrate on the gross tumor volume for fine segmentation. The work by (4) is most similar to ours as they also employed ensemble model based on multi-scale sampling, however they employed a projection block and attention block to improve the extracted representation. The projection block is similar to the popular “SqueezeExcite” (7) method used to improve the learned representation. However, in this case they squeeze the feature maps across the three dimensions which they later combined *via* summation operation across the spatial dimension and finally a projection to the depth dimension which recovers the original shape of the feature map. The attention module is a spatial attention block that focuses and refines extracted representation especially for very small tumors which is common in NPC. Despite the addition of more sophisticated blocks, we find their method under performs compared to purely using the UNet-2.5D which uses much fewer learning parameters. Although the work by (6) used magnetic resonance images in contrast to CT scans, they demonstrated that by combining the T1-weighted (T1W) and T2-weighted (T2W) MRI images of each patient provides significant performance boost. These two sequences were combined by their proposed dense connectivity embedding, which essentially fuses the feature maps of each modes across the layers in the encoder. Furthermore, a convolutional block is introduced to process the fused embedding which will then act as a skip connections to their corresponding decoder block in a UNet architecture. While MRI would instinctively be the better imaging modality to become the basis for auto-contouring, MRI is not always readily available in all countries, especially developing countries.

The use of deep learning in medical imaging has become a popular alternative for practitioners to automatically generate accurate target delineation. Furthermore, it does not only resolve the time-consuming and tedious task of manual contouring but can also alleviate the problem of inter-observer variability by generating more robust predictions since it learns from different sources. This problem occurs when radiation oncologists disagree on the delineated gross tumor volume brought by the inherent subjectivity of the annotation process itself. This depends on variety of factors notably years of experience of the practitioner. However, though deep learning models have the potential to generate significant benefits in the medical imaging field, it is also a poor field to apply these methods to. This is because data in this field are notoriously difficult and expensive to collect. And when this is coupled with the fact that deep learning models are only as good as the quantity and quality of the data you have, then the objective is to not only generate accurate models but also models that can perform well when there are few data. To this end, we employ self-supervised learning (SSL). SSL method has become the mainstream approach in mitigating problems regarding data scarcity when utilizing deep learning in the medical setting. It is able to leverage unannotated scans by using a predefined pre-text tasks (self-supervision task) which is used to train a feature-extractor or the encoder network. Ideally, this pre-text task should be able to help the encoder network or the feature extractor learn features and representations such as the generic structure, texture, and other salient features that can be re-used during the *downstream task* or the actual target task which is in our case the segmentation of GTV in NPC. Hence it will require much fewer annotated data during the downstream task making it data efficient. For our work, we used an equal number of unannotated and annotated NPC CT scans. In contrast with other works which used single pre-text task during SSL pretraining we used multiple pre-text tasks to pre-train our encoder network. Specifically we use a combination of relative-positional location (RPL) and rotation methods to pre-train our encoder network. This encoder network can then be frozen and attached to a decoder network used for the segmentation task. The goal is that by employing SSL pretraining, the feature extractor will be in a much better starting position to easily learn the diverse morphologies and sizes of the gross tumor volume even with much fewer data.

Self-supervised learning in medical images (8–14) is usually an extension of the self-supervised techniques used in 2D natural images. The seminal work by (15) proposed different pretext tasks for 3D medical image that were originally based on 2D images. Multiple pretext tasks specialized for 3D medical images are proposed such as: contrastive predictive coding, rotation prediction, jigsaw puzzles, relative patch location, and exemplar methods. The predictive coding pretext task first divides an input 3D cube into smaller cubes which are individually encoded by the network. Given a set of consecutive encoded cubes, the network must find and choose the next consecutive cube out of a set potential cubes based on their encoding. Hence, in order to accomplish this task, the network must be forced to learn the specific fine-grain morphology and structure of the volume in order to correctly predict the next adjacent cube. And because this uses contrastive learning (16), the encoding or representation of adjacent cubes are much closer than cubes that are farther away. This conforms with the actual input volume where adjacent volumes have very similar features. The important consideration here is that the network was able to learn and distinguish the feature, structure and morphology of the volume even without labels by doing this pretext task. This is essentially the same case with all pretext tasks, for rotation it randomly rotates the volume from a predefined class of orientation, then the network must predict the specific orientation but in order to correctly predict the orientation it must understand the structure of the volume. For relative-patch location, it randomly crops an input volume then divides the volume further into 27 non-overlapping cubes. It then uses the central cube to predict the location of a cube randomly queried which has a total of 26 possible locations or classes. In our work we employ the relative patch location and rotation pre-text tasks for our proposed SSL since they are much simpler and were able to produce significantly higher performance over the other pretext tasks. More recent work by (17) proposed a spatially guided self-supervised clustering network (SGSCN) for downstream medical image segmentation. They proposed using multiple loss functions to train a network in an end-to-end manner in order to group image pixels that are spatially connected and thus have similar representations. In addition, a context-based consistency loss is used to better learn the boundaries and shape of the target volume. Finally work by (18) proposed the use of auxiliary tasks for task-level consistency as an SSL approach. Specifically two auxiliary tasks are used where one task is responsible for foreground-background reconstruction aimed for in-formation segmentation while the other task employs a mean-teacher architecture to perform signed distance field (SDF) prediction to enforce shape constraints. All these SSL methods were proposed mainly to address the limited availability of labeled data while exploiting abundance of unlabeled data. Similar to ours we propose an SSL approach that uses a combination of pretext tasks to help a feature extractor learn representations from unlabeled dataset that are highly relevant to its downstream segmentation task.

Filipino oncologists have always been aware of the high number of cases of nasopharyngeal carcinoma in the Philippines based on their individual experiences in their own clinics and hospitals. The true number cannot be verified because of the absence of a government-run nationwide cancer registry. Because of the number of patients with nasopharyngeal carcinoma at our institution and the consequent volume of imaging data, we felt that it would be a good venue for the creation of auto-segmentation/auto-contouring software for radiation oncology use. Moreover, since modern deep learning methods are notoriously hungry for labelled data, we introduce a self-supervised method to compliment the development and training of our proposed deep learning method. This will mitigate overfitting introduced due to very few labelled data thereby improving performance as well as allows it to exploit unlabeled data which are often much more abundant than labelled data. This cuts costs in terms of the resources and time required to label more data to improve model performance.

Collaboration between researchers from the Department of Computer Science of the College of Engineering and the Division of Radiation Oncology of the Department of Radiology of the UP-Philippine General Hospital resulted in this study that set out to create a software that could accurately contour nasopharyngeal tumors on appropriately acquired CT scan images.

## 2 Methods

### 2.1 Network architecture

**Figure 1:**
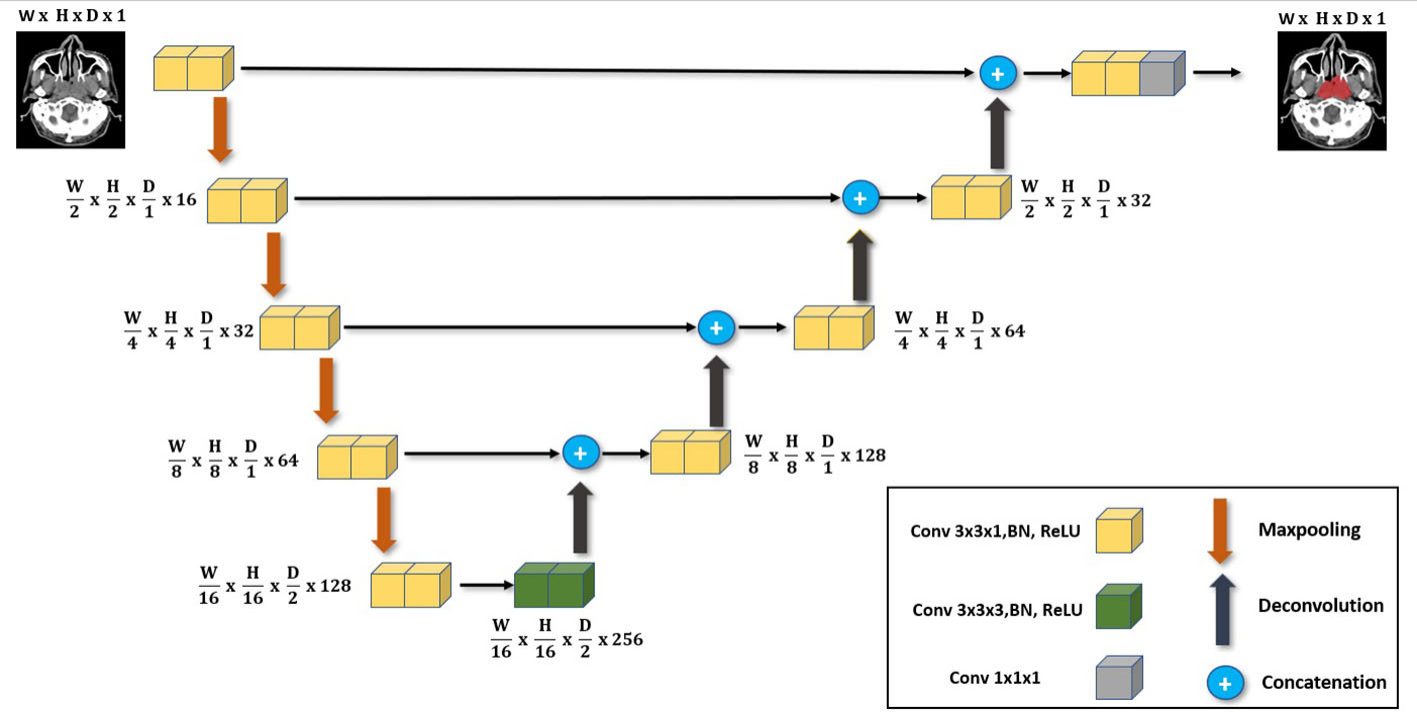
The main deep neural network architecture used in our work. It follows the same UNet architecture but with the main use of 3x3x1 convolutions instead of 3x3x3 convolutions employed for 3D volume segmentation. We highlight its effectiveness when used in data-scarce setting as it is less likely to overfit.

The primary network architecture used in this work is the UNet-2.5D (4) network based on the UNet3D (19). This is shown in **Figure 1** where the main difference compared with UNet-3D lies in the 2D convolutional block that UNet-2.5D uses. Following (19) our architecture consists of nine convolutional blocks where each block consists of two convolutional layers interleaved with Batch Normalization (20) and RELU non-linearity (21).

The difference between UNet-3D and UNet-2.5D is the dimension of the convolutional layer. UNet-3D uses 3D convolutional layer across its block whereas UNet-2.5D utilizes 2D except for the center or bottleneck block which uses 3D convolution. In general, the performance of a model is better with higher number of parameters and convolutions. However, this is not the case as we will show in our results. The simpler and lighter UNet-2.5D network – in terms of number of parameters and operations – significantly outperforms the UNet-3D network. This is because heavier networks such as UNet-3D require more data as there are more parameters to train.

In a setting where limited data are available such as our case (i.e., NPC CT images), the parameters will easily overfit the training data making the model unable to generalize its prediction to test data.

### 2.2 Multi-scale training

In general, training time is proportional to the size of the data fed to the model before it converges. In our case, the GTV in NPC is smaller relative to the entire patient’s body. Hence instead of feeding the entire volume as input to the model, we cropped the input volume along the x, y, and z directions as suggested by (4) using multiple scales encompassing the GTV. Five scales are extracted to generate five datasets. These are *extra-small*, *small*, *medium*, *large*, and *extra-large*. The smallest scale is randomly cropped across x, y, and z direction to extract a volume that contains the smallest spatial resolution and depth. By extracting the smallest volume, we ensure that we extract only the local information of the structure and feature of that given volume. The largest scale, on the other hand, captures almost the entire volume with the information extracted mostly globally.

**Figure 2:**
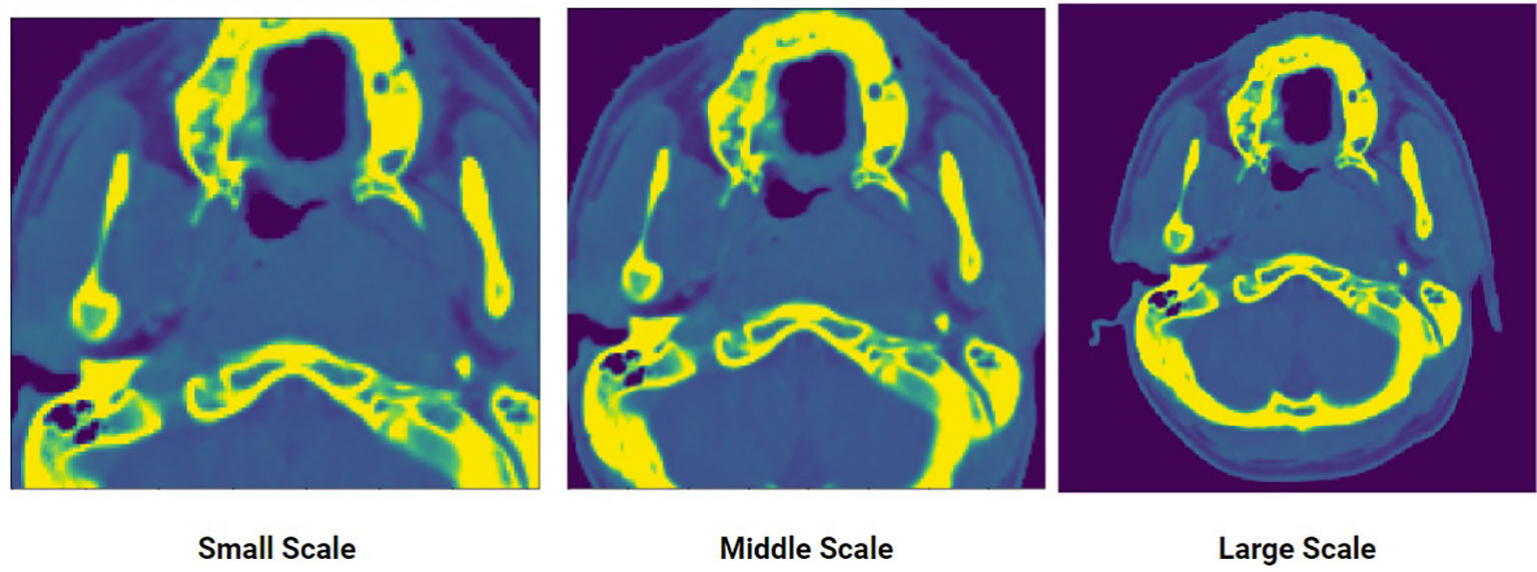
An input CT scan showing three different scales of the same scan: small, middle, large. Five scales were generated to create the multi-scale training data.

The crop-size used to extract the data for each scale decreases in a fixed percentage as the scale decreases from extra-large to extra-small. Along the z direction, the length (number of slices) of the original input volume is cropped beginning at 90% with constant decrement of 10% as the scale decreases. For the x, y dimension, the patch for the large-scale starts at 100% of the resolution (i.e., 512 x 512 pixels) then cuts with decrement of 15% as the scale decreases. This can be seen in **Figure 2**. Each of the five datasets generated is then used to train a corresponding model.

During testing, the outputs of the five trained models given the same input are aggregated to produce a single result. Empirically, this produces a much more robust result compared to simply using a single model because each model is specialized to a certain scale. Since the features of NPC varies widely in terms of size and shape, models trained using the small-scale and large-scale datasets perform better in detecting tumors that are small and large, respectively.

The rationale behind using multi-scale training is specializing each model to a certain feature or context of the volume. As it will be shown, this approach achieves significantly superior results compared to training a single model.

### 2.3 Ensemble of models

Five models were trained using the five scaled dataset obtained *via* multi-scale cropping: extra-small, small, medium, large, extra-large. During the evaluation/testing stage, we used a fixed and uncropped raw CT input scans to evaluate the performance of each of the five models. Each of the models was to make a separate prediction in the form of probability maps. To create a model ensemble, the probability maps from all the five models for a specific input are averaged to produce a single probability map. This will be used to create the final segmentation mask.

Using this model ensemble approach produces a more robust prediction. Moreover, model ensembles also boost model performance compared to using a single model. This is due to having richer and more diverse predictions from each model that is specialized to a specific scale. The drawback of this approach, however is the higher computer and memory requirements. To mitigate this, the UNet-2.5D is used since it only uses a single 3D convolution with 2D convolution for the rest of the layers. This architecture is much more lightweight and less computationally intensive than 3D convolutions

### 2.4 Semi-supervised pretraining

#### 2.4.1 Pretext tasks for self-supervised learning

**Figure 3:**
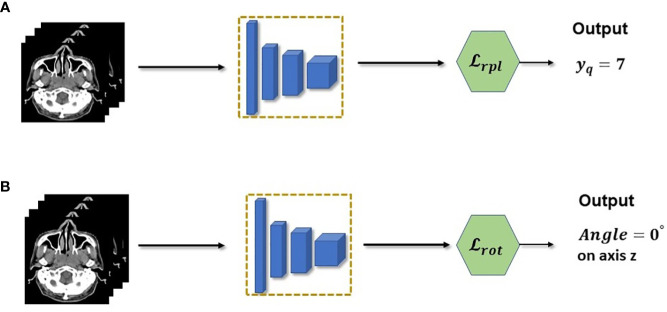
**(A)** Relative patch location is a pretext task used to pretrain the feature extractor. In practice, the task is a multi-class classification which predicts the location of a query patch. **(B)** Rotation pretext task is also casted as a multi-class classification but with rotations as the class.

The main pretext tasks used in this work are shown in **Figure 3** which were introduced by (15), these are relative patch location and rotation pretext tasks. The rotation pre-text task shown in **Figure 3B**, is one of the simplest pretext tasks and therefore can easily be implemented in any setting. The goal of the rotation pretext task is to simply predict the angle of rotation for an input data that was rotated for a specific angle. We fix the possible angles of rotation to 0°, 90°, 180°and 270°. Since there are three axis of rotations, there will be a total of 10 possible angles (since 0°is redundant for the three axis) for an 3D input image. Hence this pretext task is essentially a multi-class (10 classes) classification task where each class consists of a particular rotation angle for a specific axis. The goal is by predicting the 3D rotation of each volume, the encoder network will be forced to learn the structure of the volume and hence relevant features that can be re-used when making downstream segmentation tasks. However due to its simplicity, the features learned at convergence of the rotation pretext task may not be enough in providing the necessary features for the downstream segmentation task.

To mitigate this insufficiency, we combine the relative patch location (RPL) pretext task shown in **Figure 3A**, which consists of predicting the location of a query patch relative to a fixed anchor patch. This self-supervision task enables the model to learn a much richer structural and finer grained information within the data. This is crucial for 3D segmentation task since it needs to understand the structure and spatial features of the input to correctly predict each voxels. Discretely, the RPL pretext task is implemented by dividing a 3D input image into a 3×3×3 grid to create a total of 27 non-overlapping patches {*x_i_*_∈{1_ *_,…,N_*_}_}. The central patch *x_c_* will be used as the fixed anchor patch and a query patch *x_q_* will be randomly sampled from the remaining set of patches {{*y_n_*}. The pretext task trains an encoder model to learn the location of the query patch with respect to the central patch by predicting a location *y*ˆ*_q_*. Since there are total of 26 patches (central patch is excluded), the encoder will be trained using a multi-class (26 classes) classification similar to the rotation pretext task. In this case it is predicting the class location instead of the angle of rotation. However, it is different in that it needs to fuse both the query and anchor patch together and make the location prediction based on this fused information. This is further shown in the following equation:


(1) $${ℒ}_{RPL}=-\sum_{k=1}^{K}\log p(y_{q}|{\hat{y}}_{q},\left\{y_{n}\right\})$$

where *y_q_* corresponds to the groundtruth location of the patch.

#### 2.4.2 Combining pretext tasks for richer representation

**Figure 4:**
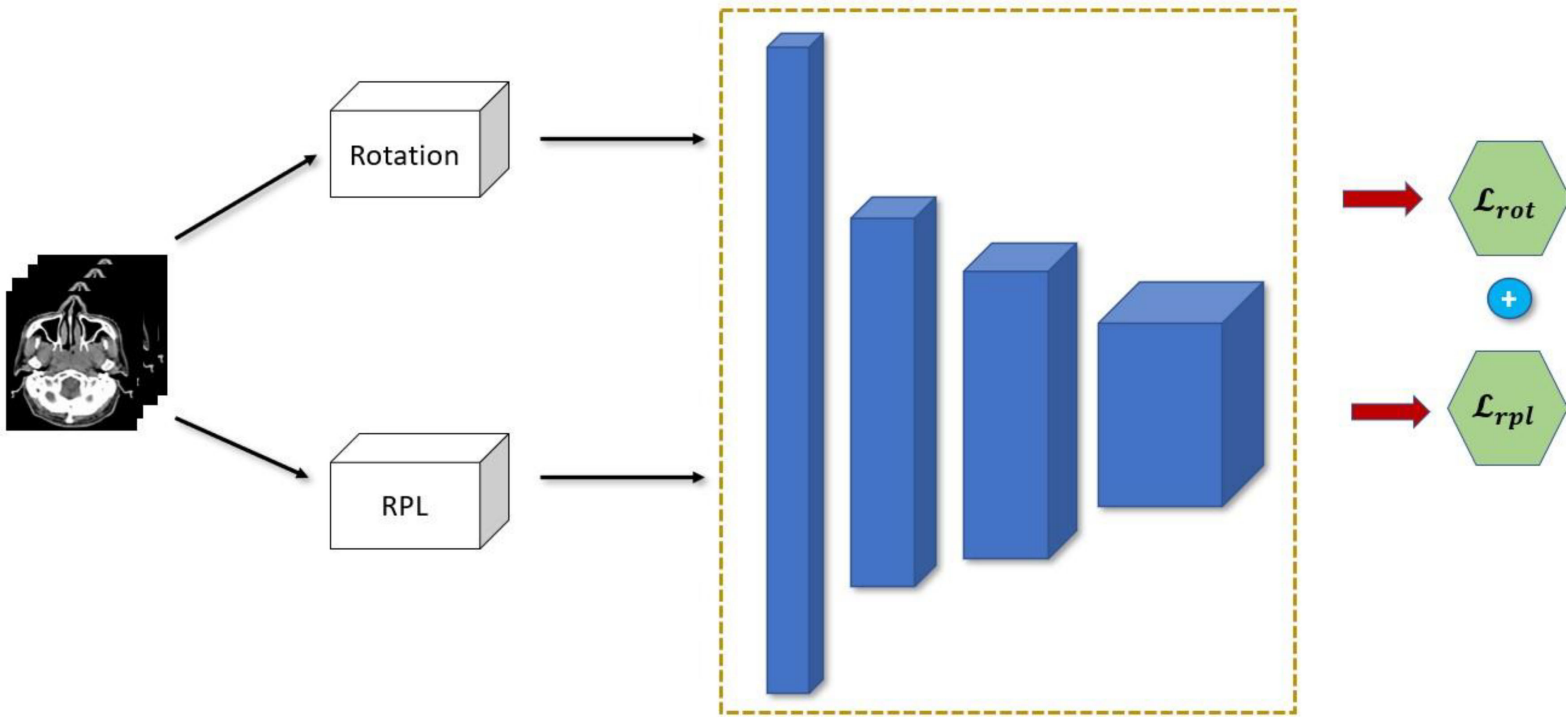
Proposed combination of semi-supervised learning pre-text tasks.

We combined the two pretext tasks shown above in order to force the encoder to learn a synergy in the feature representation that is extracted from each image. This is because the encoder needs to learn how to combine, segregate and choose the representations that are most relevant for the two tasks. And since each pretext task have varying objective, the representation should be compact and sufficient for the two tasks. Moreover, by using two pretext tasks simultaneously, the encoder will need to learn rich and diverse representations that will be much more useful for the downstream task. Since this is purely self-supervised, training our encoder network is much more data and parameter efficient. This is because it can leverage the use of unlabeled CT scans while using lighter network. The schematic of our SSL approach is shown in **Figure 4** where each image is fed to two pre-processing blocks before being fed simultaneously to the encoder network. The overall loss function therefore is shown below:


(2) $$L=\alpha L_{RPL}+(1-\alpha )L_{Rot}$$

where *α* is the weighting factor to balance and control the contribution of each pretext task.

#### 2.4.3 Efficient model ensemble

**Figure 5:**
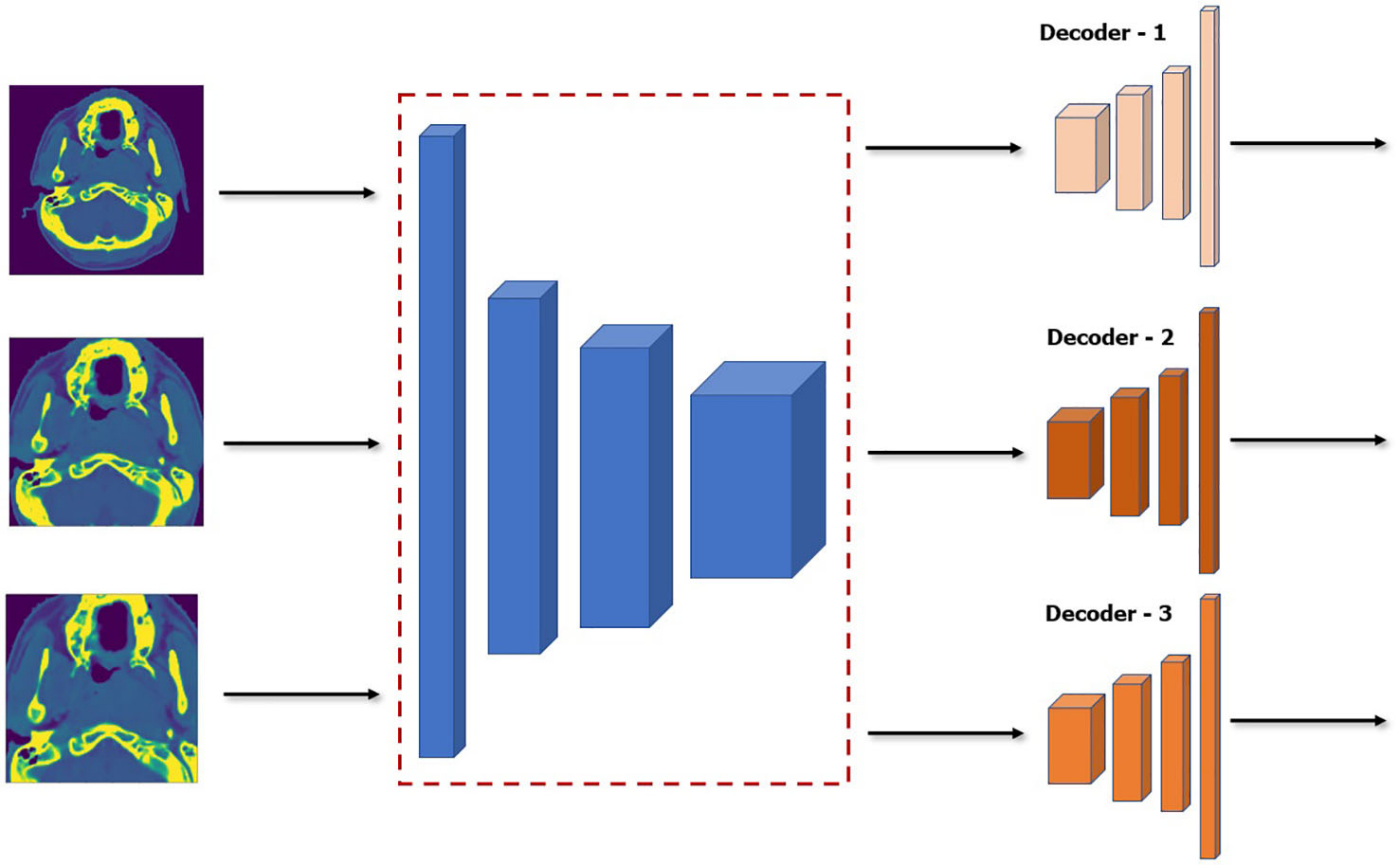
Architecture of model ensemble with a single encoder network while finetuning three decoder networks. Note that the encoder is frozen and thus will not be affected during finetuning.

Although model ensemble have been very effective in improving the performance and robustness of the model by relying on independent weak learners in traditional machine learning, it is usually impractical to use it directly in deep learning. As was discussed above, to create the model ensemble, five models are trained on five different scales of the dataset which generates five trained models. This can be computationally prohibitive especially in very deep network which can be more expensive than the performance boost it provides. Our proposed SSL approach can help mitigate this since we can essentially *freeze* and *re-use* the encoder network that was pretrained during the SSL. We can have essentially a single unified encoder network while only training or finetuning the decoder of each model in the ensemble. A diagram of this approach is shown in **Figure 5**. This makes our method much more parameter efficient during both training and inference.

## 3 Materials and methods

### 3.1 Clinical material

At the outset, it was decided that we would attempt to create an auto-segmentation or auto-contouring program using nasopharyngeal tumors. With nasopharyngeal tumors, the tumors are confined to a single anatomic space and, there is no need to account for movement, swallowing and breathing. Additionally, nasopharyngeal cancers are relatively common in the Philippines, making this work impactful.

A review of census of nasopharyngeal cancer patients at the Division of Radiation Oncology was done, covering May 2017 — when operations of the linear accelerator started — until February 2020 — just before the start of the COVID lockdown. A total of 79 patient records were retrieved. Patients who were less than 18 years of age and had non-carcinoma tumors, i.e. lymphomas, sarcomas, were excluded from consideration. The images of the remaining 63 patients — 44 males and 19 females ranging in age from 18 to 73 and covering all tumor stages — were used in this paper. Individual patient consent was waived because of the use of just the images and the retrospective nature of this study. A total of 50 healthy patients were also collected to be used for the semi-supervised pretraining of the encoder network.

Shown in **Table 1** are the baseline characteristics of the 63 NPC patients included in this study. Fifty-three (53) of these patients were randomly selected to be used in the training and validation of our models. The remaining ten (10) patients were utilized during testing. Majority of patients included in the study were male. More than half of patients had T4 disease based on the American Joint Committee on Cancer (AJCC) Cancer Staging Manual Eight edition for nasopharyngeal carcinoma.

**Table 1:** Demographic characteristics of the NPC patients included in the study.

Characteristics	Total number of patients n = 63
Median age (range)	45 (18 – 73)
Sex	
Male	44
Female	19
T classification	
T1	6
T2	10
T3	14
T4	33

Simulation computed tomography (CT) images with contrast were acquired using a SOMATOM Emotion 16 (Siemens Healthineers). All patients were positioned in supine and immobilized using a head, neck, and shoulder thermoplastic mask. Scanning range was from vertex to carina. Obtained CT images were reconstructed using a matrix of 512 × 512 with thickness of 3.0 mm. Delineation of the primary gross tumor volume on CT images was then performed by an experienced radiation oncologist. The contoured images — all in DICOM format — were anonymized before being subjected to computer “training.” Ten (10) image sets were randomly chosen and used initially for testing. The remaining fifty-three (53) image sets were used in the training and validation of the software model. For training to commence on these images, these had to be in a suitable format to be processed by the proposed deep learning model. The array volumes (3D tensor) were extracted from the DICOM files, ensuring isotropic resolution. A uniform resolution of 1.0 ×1.0×3.0mm^3^ was enforced. The Hounsfield Units (HU) of all images (originally ranging from -1024 to 3071) were truncated and normalized to [-150, 500]. All values above and below this range were set to zero.

### 3.2 Data preprocessing and augmentation

#### 3.2.1 Data preprocessing

The actual raw data that is frequently used by radiation oncologists are in a DICOM (22) format and while it is extremely useful in their specialized software tools, it cannot be directly processed by our deep learning model. It needs to be cleaned and transformed to a suitable format for training and inference. The first step is the extraction of the array volume (3D tensor) from the DICOM files and ensuring an isotropic resolution. Although the thickness of each patient’s CT scan are all 3.0 mm, the x,y pixel spacing range varies from patients to patient. We therefore enforce a uniform resolution of 1.0 ×1.0×3.0mm^3^ for the x, y, z spacing by uniform interpolation. Afterwards, since the raw array values of the DICOM files are in Hounsfield unit which ranges between -1024 and 3071 HU for each voxel, we truncate and normalize their values. We find that for body and NPC, they have a distinct distribution of Hounsfield values. We therefore truncated the Hounsfield values to [-150,500] where outside these range are all automatically set to zero. This are then finally normalized to [0,1].

#### 3.2.2 Data augmentation

Since we have very little training data, there is a high chance that the model may overfit hence we employ different data augmentation techniques that can increase data samples and thus improve model performance. We employ the most effective data augmentation techniques: rotation, flipping, cropping and transposing which are randomly applied across the x,y,z dimension for each batch size iteration during training. Flipping is also especially important as observed by (23), which highlighted that it improves the model’s robustness on different tumor shapes and which is especially important for our use case because NPC has many different shapes.

### 3.3 Evaluation metrics

There are a total of eight evaluation metrics used in the experiment, the primary performance evaluation metric and most commonly used ones are the Dice-Similarity Coefficient (DSC) and Intersection-OverUnion (IOU) metrics. Both metrics measure the same overlap between the groundtruth and the predicted mask and have a range between 0 and 1 where 0 means totally no overlap and 1 means perfect overlap. Though it may be tempting to view both metrics functionally equivalent, their distinction arises when taking their average values across set of samples. Specifically, IOU score penalizes wrong predictions much more than DSC. Thus IOU score can be thought of as measuring the lower bound of the model performance while the DSC measures the average model performance across the test data. We can expect therefore that IOU usually outputs a significantly lower score compared to DSC. This is highlighted later in the section.

The two other metrics are grouped under the distance metric which measures the distance between two sets that contain point coordinates from both the groundtruth and points predicted by the segmentation model. These metrics are the Average Symmetric Surface Distance (ASSD) and the Hausdorff Distance. The ASSD determines the average difference (24) between the surface of the predicted and groundtruth volumes. The surface points from both the prediction and groundtruth surfaces are sampled from a set of points that are not part of a predefined neighborhood. These points can be thought of as the outlier or the gap with respect to the groundtruth surface. The closest distance of each of these outlier points are then taken against the points in the other surface. The average distance of these points will be the ASSD which will be in a mm unit. ASSD score will be 0mm for perfect segmentation, with increasing score corresponding to worsening performance of the model. The Hausdorff Distance is similar to the ASSD except that it does not measure the average distance between outliers of two surfaces, but rather measures the maximum distance of randomly samples points from the two volumes to create two sets. The Hausdorff Distance is then the maximum distance from a point in one set to the closest point in the other set. Again the lower the distance, the closer the points between the groundtruth and predicted volumes are.

The final four metrics are: sensitivity, relative volume error, and positive predictive value (PPV). These are the most commonly used metrics for medical image segmentation in deep learning. The sensitivity, also referred to as true positive rate quantifies the model’s ability to correctly detect the voxels that is indeed an NPC or tumor. It measures the proportion of voxels in the volume that are truly tumors and are correctly detected by the model. Finally the PPV is simply the ratio of voxels that were correctly identified as tumors to the voxels that were identified to be tumors. Or essentially it is the probability that the voxels that were predicted as tumors are indeed tumors.

### 3.4 Post-processing

Post-processing involves the aggregation of the individual model prediction in the ensemble and a heuristic-based post-processing to further refine the prediction. It has been observed that the aggregated output from the model ensemble still have some residual volumes that are sparsely distributed and are not attached from the largest volume prediction. Since it is assumed that we are only predicting the primary tumor volume, the final prediction should only have a single large solid volume. Hence we first perform a series of morphological operation (i.e., erosion and dilation) to remove the edges in the volume. Afterwards, for each 2D-slice of the volume, a contour search is applied to get only the largest 2D contiguous mask and removing the remaining 2D contours. This operation is applied across all the slice in a volume essentially taking only the largest connected region as the final volume prediction.

### 3.5 K-fold cross validation and implementation details

Since there are a total of 63 patients, we performed 7-fold cross-validation where 54 patients are used for training and validation and the remaining 9 patients will be used for evaluation for the final model. The final performance is averaged across the seven folds. During the training run, training validation data for each fold is split 80/20 respectively, where the validation is used to tune the hyperparameters. After finding the optimal hyperparameters, the training and validation data are combined to generate a final model which will be evaluated on the test dataset.

All the experiments were implemented using the Pytorch deep learning framework using NVIDIA RTX 2080Ti Graphical Processing Unit (GPU) 11GB. The ADAM (25) optimizer was employed to train our deep learning network using an initial learning rate of 1 × 10^−3^ and with a decay factor of 1 × 10^−4^ for every 150 epochs. The whole training-validation run takes a total of 900 epochs using 32 batchsize for each iteration. Moreover, random cropping is done where volume of patches is randomly extracted from each patient volume and fed to the network. This approach mitigates the memory constraint in the GPU and speeds up loss convergence. This is applied for the whole five-scaled dataset to generate five pretrained models for inference and testing.

## 4 Results and discussion

### 4.1 Method comparison

We evaluate first our proposed approach(UNet-2.5D) against different architectures commonly employed for medical image segmentation. The other architectures tested are UNet-3D, VNet and the UNet + Project Excite(PE) + Attention Module(AM) by (4) proposed specifically for the segmentation of GTV in NPC. We show that with the simpler UNet-2.5D architecture, it significantly outperforms the generic UNet architectures as well as the network proposed by (4).

Moreover, we compare our method on popular architectures that has gained state-of-the-art performance on multiple benchmark dataset. One is the Generic Autodidactic Models or Genesis model proposed by (26). The Genesis model aims to provide a generic source model that can be transferred on different application-specific target task. It achieved broad performance improvement over different medical segmentation benchmark dataset from chest to brain data. In our case we use their pretrained UGenesis model trained on chest CT as our base model then finetune it to our dataset. Another very popular method that achieved multiple SOTA results is the no-new-Unet or nnUNet by (27). They have shown that for a fully optimized network, “architectural tweaking” provides no improvement in the segmentation performance, and the influence of non-architectural aspects in segmentation methods is much more impactful. nnUNet offers an end-to-end automated pipeline that is adaptable to any medical dataset. It has an automated pipeline for preprocessing, data augmentation, and post-processing. It can also automatically infer important hyperparameters such as normalization, resampling and batchsize optimized for the given dataset. For our case, we employ the nnUNet for all the three available architecture types: 2D, Fully 3D and Low Resolution 3D. We use the same seven-fold cross validation for all the evaluation runs.

Except for nnUNet, all the different segmentation methods made use of the *medium-scale* preprocessed data as their training set. This is because data preprocessing from raw data is part of nnUNet’s automated pipeline.

The quantitative results for DSC, IOU, PPV and RVE are shown in **Table 2**. These values are the average value (and standard deviation) from the seven fold cross-validation discussed above. Results show that UNet-2.5D network generally outperforms the other methods except in PPV. Since the bulk of the convolutional blocks used in our network is 2D convolutions, this may suggest that for the segmentation of gross tumor volume in NPC, the across-slice or depth-wise information does not really improve the performance. This also means that the 2D spatial information is more than enough to achieve high predictive performance. Moreover, it seems adding 3D information in predicting each voxel may actually hurt the segmentation performance as shown in VNet and UNet-3D architectures. This may be due to the structural characteristics of the NPC tumor itself, which has a random and irregular tumor structure. Aside from not adding any performance benefits, the added parameters using 3D convolution will only hurt performance because of overfitting. This makes the proposed approach not only much more powerful in segmenting NPC tumors but more efficient as it mostly uses 2D convolution with a single 3D convolution at the bottleneck region of the network.

**Table 2:** Comparative result of different deep neural network architectures for DSC, IOU, PPV and RVE.

Method	DSC (%) ↑	IOU (%) ↑	PPV (%) ↑	RVE (%) ↓
UNet-3D	66.01 ± 5.29	43.54 ± 3.49	86.03 ± 6.89	55.14 ± 4.42
VNet	64.25 ± 7.06	46.74 ± 5.13	70.23 ± 7.71	59.55 ± 0.86
UNet-2.5D+PE +AM	67.54 ± 2.16	51.15 ± 1.63	90.32 ± 2.87	38.21 ± 1.22
UGenesis	58.30 ± 7.31	41.68 ± 5.22	83.35 ± 10.44	45.24 ± 5.67
nnUNet-2D	63.14 ± 5.52	52.68 ± 4.61	63.69 ± 5.57	12.57 ± 1.10
nnUNet-3D Full	65.50 ± 8.43	54.65 ± 7.03	66.01 ± 8.50	13.05 ± 1.68
nnUNet-3D Low Res.	66.22 ± 7.94	55.25 ± 6.63	66.80 ± 8.01	13.19 ± 1.58
UNet-2.5D (Ours)	72.47 ± 4.10	60.46 ± 3.42	73.09 ± 4.14	14.43 ± 0.82

↑ means that higher means better while ↓ symbol means lower is better.

Although the method proposed by (4) was able to achieve the highest PPV in **Table 2**, the addition of Projection-Excitation and Attention-Module blocks did not significantly achieve high performance on the other metrics.

Our method was also compared on other nnUNet and UGenesis family which were all outperformed by our method. The nnUNet “3D low resolution” variant was able to achieve the highest DSC score but generally under performed in relative to even the generic networks. UGenesis with the use of a pretrained model significantly underperformed across all the metrics. This is probably due to overfitting as the number of parameters and network architecture of UGenesis is much deeper.

Results for ASSD, Hausdorff distance and sensitivity for the different architectures are shown in **Table 3**. Compared to **Table 2**, our method was only able to decisively outperform other methods in the sensitivity metric. The highest performance for the ASSD and Hausdorff metrics were achieved generally by the nnUNet family although our method is still relatively competitive especially in ASSD metric where our method is statistically equal when taking into account their standard deviation.

**Table 3:** Comparative results using distance metric as another measure between predicted and groundtruth contours.

Method	ASSD (mm) ↓	Hausdorff (mm) ↓	Sensitivity (%) ↑
UNet-3D	7.55 ± 0.61	27.83 ± 2.23	59.56 ± 4.77
VNet	7.84 ± 0.86	33.28 ± 3.65	50.61 ± 5.45
UNet-2.5D+PE +AM	5.42 ± 0.17	25.52 ± 0.81	55.87 ± 1.78
UGenesis	6.15 ± 0.77	25.61 ± 3.21	49.86 ± 6.25
nnUNet-2D	3.31 ± 0.28	14.58 ± 1.27	63.91 ± 5.59
nnUNet-3D Full	3.43 ± 0.44	15.12 ± 1.95	66.29 ± 8.83
nnUNet-3D Low Res.	3.47 ± 0.42	15.29 ± 1.83	67.03 ± 8.04
UNet-2.5D (Ours)	3.79 ± 0.21	16.73 ± 0.94	73.35 ± 4.15%

↑ means that higher means better while ↓ symbol means lower is better.

### 4.2 Ensemble results

As discussed above in order to create a more robust, less data-scale dependent model as well as to boost performance, we generated five versions of the training dataset with different scales and generated five models to create an ensemble of model. We used our proposed architecture for the architecture of all the five models which we have established to be superior on majority of metrics in **Tables 2**, **3**. These five models constituted the model ensemble. The performance of each model in the ensemble is shown in **Table 4**. As shown, models have different performance across different data scale. Notably the model trained with medium-scale outperformed the rest of the models including the aggregated ensemble performance for the DSC and RVE metrics, while the model trained on extra-small scale data achieved the best performance for the IOU metric. This means that some data scales offer the optimal information for different metrics, such as tumor’s structure, topology and texture which are more likely to be emphasized in a specific data scale. The optimal inference therefore can be obtained by averaging and combining the predictions of the five models. In a way by coming the predictions, the voxel tumor that were missed by one model because it was trained on small scale dataset may be found by model trained on the large-scale dataset. This is very useful especially in the case of NPC segmentation where the GTV have diverse morphology and sizes. This mimics a kind of majority voting for a specific voxel across the models which makes it much more robust. This also offers a kind of confidence for the model prediction. Furthermore, this allows us to measure uncertainty of model prediction.

**Table 4:** Model ensemble performance for DSC, IOU, PPV and RVE, where each data scale corresponds to a separate and unique model trained on that specific data scale.

Model ensemble performance				
Data-scale	DSC (%) ↑	IOU (%) ↑	PPV (%) ↑	RVE (%) ↓
Extra-Small	69.85 ± 4.06	61.23 ± 3.56	74.18 ± 4.31	16.56 ± 0.96
Small	72.13 ± 3.20	58.93 ± 2.62	76.32 ± 3.39	15.15 ± 0.67
Medium	72.47 ± 4.10	60.46 ± 3.42	73.01 ± 4.14	14.44 ± 0.82
Large	71.06 ± 3.31	58.12 ± 2.71	67.86 ± 3.16	26.35 ± 1.41
Extra-Large	66.41 ± 6.44	54.62 ± 5.30	68.92 ± 6.68	16.10 ± 1.56
Ensemble Model	72.02 ± 4.13%	60.87 ± 3.40	74.61 ± 4.19	15.97 ± 0.83

↑ means that higher means better while ↓ symbol means lower is better.

We also evaluated the performance of each model in the ensemble for ASSD, Hausdorff distance and sensitivity. The highest performance for ASSD and Hausdorff metrics where conclusively achieved by the ensemble-model. This makes sense since most of the uncertainty and difference in segmentation occurs around the boundary of the GTV. By using the prediction of the ensemble model, the boundary predictions have more confidence (when majority of models predict that a boundary voxel is a GTV) and false positive predictions are removed (when only a single model predicts that a voxel is a GTV). Although the ensemble model was not able to achieve the best performance for the sensitivity it is still relatively close and competitive.

### 4.3 Semi-supervised learning pretraining results

As mentioned in the discussion above, we used a semi-supervised learning method through the combined *Rotation*+*RPL* pretext tasks training to generate an encoder block that can extract sufficient representation even with few data. Moreover as the encoder block is assumed to be capable to extract sufficient representation for segmentation performance we can therefore freeze the encoder block during finetuning for the GTV segmentation. This effectively means that we will only finetune and train the decoder block which is very efficient especially when employing multi-scale training for model ensemble. This is quantitatively shown in the number of network parameters that needs to be trained when using a full model compared when the encoder is frozen, as shown in **Table 5**.

**Table 5:** UNet-2.5D parameter comparison.

Network setting	Number of parameters
Full Model	3,845,058
Decoder Only (Frozen Encoder)	895,122

The number of parameters for the full UNet-2.5D network is more than *4x* the number of parameters compared to when the encoder is frozen which makes sense since the encoder or feature extractor is the backbone network. This efficiency is further increased when doing a full ensemble model as we do not need to create separate encoders across different models trained on different data scale since we can re-use the frozen encoder. Since the power of a network depends directly on the number of parameters that it can use to model the data, performance will naturally degrade if you use fewer parameters however since the encoder was pre-trained, the knowledge it gained during the pretext task is very useful and transferable during the segmentation of GTV and there might no significant performance degradation. In our case, we observed minimal performance degradation compared to the performance shown in **Tables 4**, **6**, which we performed the same exact evaluation. These results are shown in **Tables 7**, **8**. For **Table 7**, there is very small performance degradation in the model ensemble performance for DSC and RVE metrics. In fact for the IOU and PPV metric, the model ensemble performance with the SSL-trained encoder achieves higher performance albeit slight increase. Hence this method is not only much more parameter efficient but is actually on par with the performance of a full model.

**Table 6:** Distance metric results of the model ensemble for each data scale highlighting the effectiveness of the model ensemble.

Model ensemble performance with distance metric			
UNet-2.5D-Scale	ASSD (mm) ↓	Hausdorff (mm) ↓	Sensitivity (%) ↑
Extra-Small Scale	3.87 ± 0.22	21.98 ± 1.28	67.69 ± 3.93
Small	4.28 ± 0.19	27.70 ± 1.23	69.80 ± 3.01
Medium	3.79 ± 0.21	16.73 ± 0.95	73.35 ± 4.15
Large	4.64 ± 0.21	22.04 ± 1.03	77.74 ± 3.62
Extra-Large	7.07 ± 0.68	21.21 ± 2.05	65.72 ± 6.37
Ensemble-Model	4.22 ± 0.29	15.73 ± 0.89	71.43 ± 4.20

↑ means that higher means better while ↓ symbol means lower is better.

**Table 7:** Model ensemble performance for DSC, IOU, PPV and RVE using a single unified SSL-pretrained encoder hence, effectively training only the decoder block.

Ensemble performance with a frozen SSL-Pretrained encoder				
Data-Scale	DSC (%) ↑	IOU (%) ↑	PPV (%) ↑	RVE (%) ↓
Extra-Small	71.05 ± 2.32	62.28 ± 2.04	75.46 ± 2.46	16.84 ± 0.55
Small	71.68 ± 2.55	58.56 ± 2.09	75.84 ± 2.70	15.05 ± 0.54
Medium	71.76 ± 2.48	59.87 ± 2.07	72.38 ± 2.51	14.29 ± 0.49
Large	70.97 ± 3.17	58.06 ± 2.59	67.80 ± 3.03	30.32 ± 1.35
Extra-Large	65.18 ± 4.87	53.61 ± 4.01	67.64 ± 5.05	15.80 ± 1.18
Ensemble Average	71.16 ± 2.93	61.62 ± 2.44	75.59 ± 2.95	15.37 ± 0.52

↑ means that higher means betterwhile ↓ symbol means lower is better.

**Table 8:** Distance metric performance results using a single unified encoder block for multiple decoder for a specific data scale.

Distance metric performance with a Frozen SSL-Pretrained encoder			
UNet-2.5D-Scale	ASSD (mm) ↓	Hausdorff (mm) ↓	Sensitivity (%) ↑
Extra-Small Scale	3.93 ± 0.13	22.36 ± 0.73	68.86 ± 2.25
Small	5.23 ± 0.15	28.70 ± 0.98	69.36 ± 2.47
Medium	3.56 ± 0.13	15.57 ± 0.57	72.64 ± 2.51
Large	4.14 ± 0.20	20.42 ± 0.85	78.38 ± 3.47
Extra-Large	8.47 ± 0.78	20.82 ± 1.55	64.51 ± 4.82
Ensemble-Average	6.49 ± 0.37	16.22 ± 0.64	72.65 ± 3.08

↑ means that higher means better while ↓ symbol means lower is better.

The model ensemble performance of the SSL-trained encoder in the distance metrics shown in **Table 8** shows a relatively steeper performance degradation. For the ASSD and Hausdorff metrics, the full model previously achieved 4.22mm and 15.73mm respectively while the SSL-trained encoder degraded to 6.49mm and 16.22mm. However in the sensitivity metric, SSL-trained encoder outperformed the sensitivity value achieved in the previous model-ensemble, 72.65% vs. 71.43%.

Aside from parameter efficiency, a more important benefits that the SSL-trained encoder can provide is data efficiency. More specifically labelled data efficiency which means that a model can achieve a specific level of performance with only a fraction or portion of the data. This has a greater practical advantage both to the doctors and annotators. They can save much more time and effort in generating, collecting and actually annotating data if the model requires much fewer data to achieve a specific baseline performance. We test to see the effectiveness of using SSL-trained encoder in achieving data efficiency. To see this we use different portions of the labelled data for training and evaluate their DSC. Moreover we compare the performance of the SSL-trained encoder to the full model to essentially see whether the use of SSL-trained model is indeed data efficient. The quantitative results for this experiment are shown in **Table 9** which shows that an SSL-trained network with frozen encoder significantly outperforms the full model especially with very limited number of data as highlighted when the portion of labelled data is 10% and 30%, the SSL-trained frozen encoder was able to outperform the full model by 38.64% and 31.08% higher performance respectively. This shrinks as the number of data increases which is later outperformed by the full model when the full dataset is available. Again, the full model is using more than 4x the number of parameters compared to the SSL-trained network with frozen encoder. This effectively highlights the usefulness of using SSL-trained frozen encoder.

**Table 9:** The DSC segmentation performance using the medium-scale dataset for SSL-trained encoder vs. full model.

% of labelled data	frozen encoder (%)	Full model (%)	Percentage difference
20%	64.18 ± 2.35	46.29 ± 1.70	+38.64%
30%	64.95 ± 2.07	49.55 ± 1.58	+31.08%
50%	68.89 ± 2.93	61.75 ± 2.62	+11.56%
100%	71.76 ± 2.48	72.47 ± 4.10	-0.98%

## 5 Conclusion and recommendations

Nasopharyngeal carcinoma, a cancer common in Asia and Africa, is currently treated with a combination of chemotherapy and radiotherapy. To achieve precise radiation treatments, accurate target delineation is critical but not always easy, especially in the head and neck area where not only the gross tumor volume requires delineation and contouring, but also the lymph node drainage areas and the numerous organs at risk. Target delineation on CT scan images takes time, knowledge and experience. Automatic segmentation can make this task more objective and efficient.

The use of deep learning is a continuously progressing direction in advancing modern medical imaging. This work hopes to be an addition in advancing this goal. Although data scarcity has always been an issue especially in the medical field, we have been able to design and create a deep learning model that is able to perform automatic contouring of gross tumor volume of nasopharyngeal cancer (NPC).

Compared with other architectures, our proposed method is able to significantly outperform other architectures in segmenting NPC. Furthermore, our method is much more efficient as it uses only 2D convolution compared to 3D convolutions used by other architectures.

This highlights that in NPC, 2D convolution is enough and may suggest that across slice information does not only improve performance but degrades it. This may be a result of NPC’s structure and topology, in that it forms no regular pattern in its structure but are random and irregular. Hence, the across-slice information adds little information to the model during training.

Moreover, we also leverage a multi-scale training data using five different scales. This allowed us to generate an ensemble of models that is more robust than the individual model. More importantly we have employed the use of semi-supervised learning through the combined rotation and relative-patch-location pre-text tasks to pretrain and freeze an encoder network. This made it 4 times more efficient in terms of the number of parameters required as well as very data efficient. We have shown that even with a portion of labelled data we are able to reach close performance by a model trained from scratch but using all the training. This has a much greater practical usage in terms of the time and resources needed to collect and annotate the data. Moreover it allows one to exploit and take advantage of the abundant data of healthy patients. We believe for future works, that achieving higher performance with fewer data will gradually become the central focus of researchers as use of medical data tightens.

## Data availability statement

The datasets presented in this article are not readily available because of data security. Requests to access the datasets should be directed to jldomoguen@up.edu.ph.

## Ethics statement

The informed consent was waived given the retrospective nature of this study.

## Author contributions

All authors discussed and conceptualized the study. JD wrote the program and drafted the first manuscript together with JM. All authors analyzed and interpreted the data. PN and JC overviewed the study and guided the preparation of the manuscript. All authors contributed to manuscript revision. All authors read and approved the final manuscript.

## Funding

The authors would like to acknowledge Engineering Research and Development for Technology (ERDT) consortium and Philippine Department of Science and Technology (DOST) for the funding of this work.

## Conflict of interest

The authors declare that the research was conducted in the absence of any commercial or financial relationships that could be construed as a potential conflict of interest.

## Publisher’s note

All claims expressed in this article are solely those of the authors and do not necessarily represent those of their affiliated organizations, or those of the publisher, the editors and the reviewers. Any product that may be evaluated in this article, or claim that may be made by its manufacturer, is not guaranteed or endorsed by the publisher.
